# Combine Use of Selected *Schizosaccharomyces pombe* and *Lachancea thermotolerans* Yeast Strains as an Alternative to the Traditional Malolactic Fermentation in Red Wine Production

**DOI:** 10.3390/molecules20069510

**Published:** 2015-05-26

**Authors:** Ángel Benito, Fernando Calderón, Felipe Palomero, Santiago Benito

**Affiliations:** Departamento de Química y Tecnología de Alimentos, Universidad Politécnica de Madrid, Ciudad Universitaria S/N, 28040 Madrid, Spain; E-Mails: angel@urbinavinos.com (A.B.); fernando.calderon@upm.es (F.C.); felipe.palomero@upm.es (F.P.)

**Keywords:** *Schizosaccharomyces pombe*, *Lachancea thermotolerans*, malic acid, lactic acid, urea, ethyl carbamate, histamine

## Abstract

Most red wines commercialized in the market use the malolactic fermentation process in order to ensure stability from a microbiological point of view. In this second fermentation, malic acid is converted into l-lactic acid under controlled setups. However this process is not free from possible collateral effects that on some occasions produce off-flavors, wine quality loss and human health problems. In warm viticulture regions such as the south of Spain, the risk of suffering a deviation during the malolactic fermentation process increases due to the high must pH. This contributes to produce wines with high volatile acidity and biogenic amine values. This manuscript develops a new red winemaking methodology that consists of combining the use of two non-*Saccharomyces* yeast strains as an alternative to the traditional malolactic fermentation. In this method, malic acid is totally consumed by *Schizosaccharomyces pombe*, thus achieving the microbiological stabilization objective, while *Lachancea thermotolerans* produces lactic acid in order not to reduce and even increase the acidity of wines produced from low acidity musts. This technique reduces the risks inherent to the malolactic fermentation process when performed in warm regions. The result is more fruity wines that contain less acetic acid and biogenic amines than the traditional controls that have undergone the classical malolactic fermentation.

## 1. Introduction

Pasteur, at the beginning of his oenological studies, considered malolactic fermentation as something unwanted, as he viewed lactic bacteria to be wine spoilage microorganisms. Later on, it has been assumed that to perform malolactic fermentations under controlled conditions is the best and almost unique way to stabilize a red wine from a microbiological point of view. Nevertheless in the last few years it has been proved that there are other different yeast species able to consume malic acid [1,2,3,4] and also others able to produce lactic acid [4,5,6,7,8].

The presence of non-*Saccharomyces* wild yeasts in fermentations was traditionally associated with high levels of acetic acid and other off-flavours. Nevertheless, nowadays researchers and winemakers are aware of the positive influence of non-*Saccharomyces* in wine quality complexity [8]. When the main objective is to produce dry wine, the difficulty with which non-*Saccharomyces* wine yeast finishes the alcoholic fermentation requires the development of multi-starter fermentations with *Saccharomyces cerevisiae* or another high fermentative yeast species as a binding partner. Nevertheless, some of these non-*Saccharomyces* could be used alone in the production of sweet wines. Some enzymatic properties (glycosidases, β-lyase, *etc.*), ethanol reduction and the release of some interesting metabolites such as glycerol, pyruvic acid, and mannoproteins among others, are the main highlights that justify the interest in these mixed fermentations [1,9,10].

Some studies have analyzed the use and influence of different non-*Saccharomyces* species in wine quality. Some of these yeast species are *Kloeckera apiculata* [11], *Hanseniaspora uvarum* [12], *Hanseniaspora viane* [13], *Torulospora delbrueckii* [14,15,16], *Candida pulcherrima* [16,17,18], *Candida zemplinina* [19], *Zygosaccharomyces bailii* [20,21], *Schizosaccharomyces pombe* [22], *Lachancea thermotolerans* [7] and *Hansenula anomala* [23,24]. Most of these studies report sequential inoculation of a non-*Saccharomyces* and a *Saccharomyces cerevisiae* as the best option.

Among non-*Saccharomyces* yeast species, *Schizosaccharomyces pombe* has been used for deacidification purposes, due to its ability to convert l-malic acid into ethanol [25]. On the other hand, during the last years new uses of this genus have been developed [1]. One of these new uses is its application in ageing over lees, due to their polysaccharide release superiority [26]. The literature also describes the use of certain *Schizosaccharomyces* mutants to reduce the initial content of gluconic acid in spoiled grape musts [27,28,29,30,31]. *S. pombe* fermentation also provides a way of increasing the overall pyranoanthocyanin content in red wines [32,33]. Nevertheless, due to the great variability in the genetical composition of *S. pombe* [34], further selection processes must be performed [35,36] in order to obtain proper strains for winemaking. *Lachancea thermotolerans* has been recently described for acidification of low acidic musts [5,6,7].

This study demonstrates that it is possible to produce a quality wine without using the genera *Saccharomyces* and to avoid any possible collateral effects produced by lactic bacteria in wines with high pH and high alcohol content. In these cases it is very difficult to develop a proper malolactic fermentation process without any deviation. For these reasons, the combined use of *Lachancea thermotolerans* and *Schizosaccharomyces pombe* is proposed as an alternative to the classical malolactic fermentation in red wine.

## 2. Results and Discussion

### 2.1. Fermentation Kinetics

#### 2.1.1. Yeast Population Kinetic

**Figure 1:**
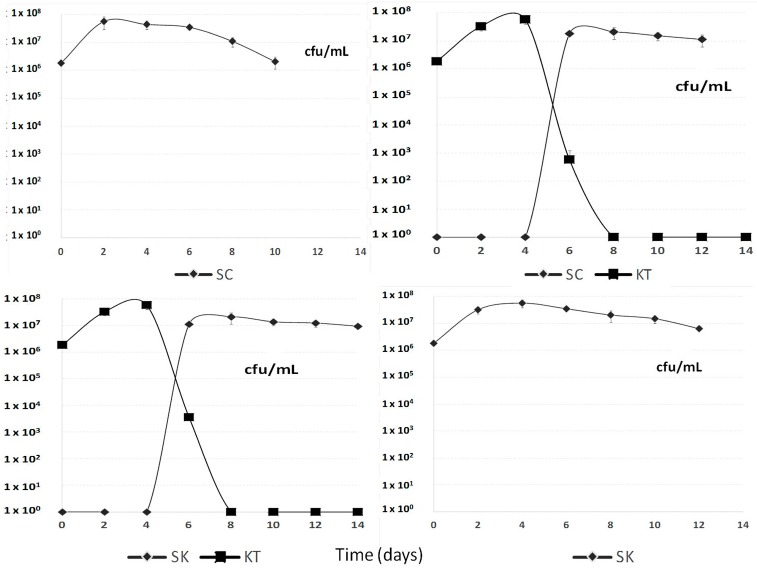
Population development of *Saccharomyces cerevisiae* 87 (SC), *Kluyveromyces thermotolerans* CONCERTO™ (KT) and *Schizosaccharomyces pombe* V2 (SK) during the different sequential fermentation processes.

Figure 1 shows the different yeast strain population development during the fermentation processes. In sequential fermentations, when *Saccharomyces cerevisiae* 87 or *Schizosaccharomyces pombe* V2 were inoculated, *Kluyveromyces thermotolerans* CONCERTO™ started to decline fast by day 4, although it was faster in the case involving *Saccharomyces*.

The early disappearance of *Kluyveromyces thermotolerans* CONCERTO™ could be explained due to presence of an ethanol concentration higher than 6% *v*/*v* by day 4, although this species has been reported to tolerate up to 9% *v*/*v* ethanol when it ferments by itself [5]. This low alcohol tolerance by *Lachancea thermotolerans*, make it impossible to produce a dry red wine in warm regions without using another more fermentative yeast in a sequential fermentation.

#### 2.1.2. Sugar Consumption Kinetics

**Figure 2:**
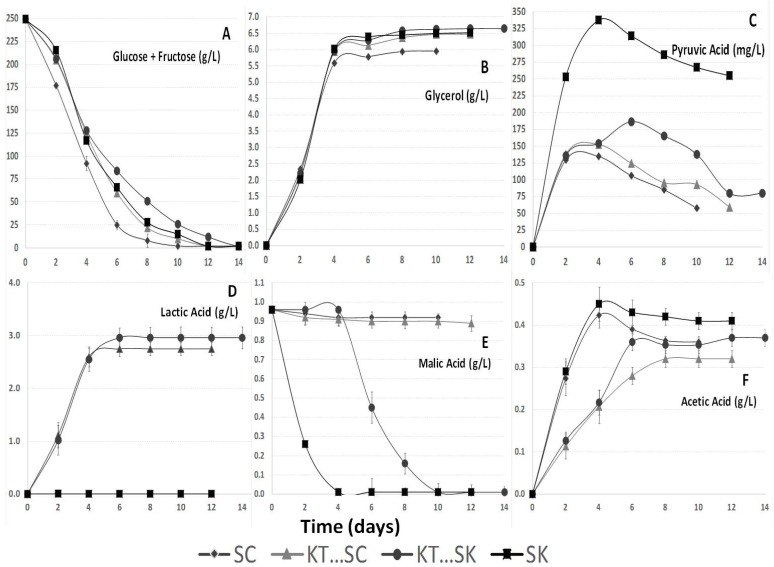
(**A**) Glucose + fructose concentrations (g/L); (**B**) Glycerol concentrations (g/L); (**C**) Pyruvic acid (mg/L); (**D**) l-Lactic acid concentrations (g/L); (**E**) l-Malic acid concentrations (g/L); (**F**) Acetic acid concentrations (g/L). Parameters of the studied wines based on Tempranillo variety during fermentations performed by *Saccharomyces cerevisiae* 87 by itself (SC), sequential fermentation with *Saccharomyces cerevisiae* 87 and *Kluyveromyces thermotolerans* CONCERTO™ (KT···SC), sequential fermentation with *Schizosaccharomyces pombe* V2 and *Kluyveromyces thermotolerans* CONCERTO™ (KT…SK), and *Schizosaccharomyces pombe* V2 by itself (SK).

The fermentations involving *Saccharomyces cerevisiae* 87 (SC) (Figure 2A) consumed the sugar the fastest. Slower glucose and fructose consumption kinetics have been described before for *Schizosaccharomyces pombe* in spite of the fact that this yeast is able to consume all sugar in a regular must [22,36]. All the studied fermentations were finished properly between days 10 and 14 reaching values lower than 3 g/L in the sum of glucose and fructose, although there were some differences between them (Figure 2A).

### 2.2. Chemical Parameter Monitoring

#### 2.2.1. Glycerol

Most glycerol was produced during the first days of fermentation (Figure 2B). The SC fermentation reached the lowest level in glycerol and KT···SK fermentation showed the highest final content. *Lachancea* and *Schizosaccharomyces* genera have been reported before as higher glycerol producers than *Saccharomyces* [7,33,37]. Final levels of glycerol varied from 5.96 g/L to 6.65 g/L (Table 1). Increased glycerol content is described as one of the main contributions of non-*Saccharomyces* strains on wine quality [38].

**Table 1:** Final analysis of *Saccharomyces cerevisiae* 87 by itself (SC), sequential fermentation with *Saccharomyces cerevisiae* 87 and *Kluyveromyces thermotolerans* CONCERTO™ (KT···SC), sequential fermentation with *Schizosaccharomyces pombe* V2 and *Kluyveromyces thermotolerans* CONCERTO™ (KT…SK), *Schizosaccharomyces pombe* V2 by itself (SK), and fermentations after malolactic fermentation with *Oenococcus oeni* 217 (+ MLF).

Compounds	SC	SC + MLF	KT···SC	KT···SC + MLF	KT···SK	SK
l-Lactic Acid (g/L)	0.01 ± 0.01a	0.54 ± 0.08b	2.75 ± 0.12c	3.27 ± 0.19d	2.96 ± 0.21c	0.01 ± 0.01a
l-Malic Acid (g/L)	0.92 ± 0.02b	0.01 ± 0.01a	0.89 ± 0.04b	0.01 ± 0.01a	0.01 ± 0.01a	0.01 ± 0.01a
Acetic Acid (g/L)	0.36 ± 0.01b	0.44 ± 0.05c	0.32 ± 0.02a	0.39 ± 0.04bc	0.37 ± 0.02b	0.41 ± 0.02c
Residual Sugar (g/L)	2.08 ± 0.30b	0.12 ± 0.04a	2.22 ± 052b	0.16 ± 0.04a	2.41 ± 0.58b	2.13 ± 0.17b
Glycerol (g/L)	5.96 ± 0.02a	5.89 ± 0.05a	6.48 ± 0.05b	6.36 ± 0.06b	6.65 ± 0.04c	6.59 ± 0.03bc
Free SO_2_ (mg/L)		26.12 ± 2.38a		25.25 ± 3.43ab	25.25 ± 3.28ab	21.15 ± 1.28b
Total SO_2_ (mg/L)		56.52 ± 2.43b		44.13 ± 3.16a	46.50 ± 3.21a	58.58 ± 1.15b
Alcohol (% *v/v*)	14.56 ± 0.01c	14.54 ± 0.02c	14.20 ± 0.04b	14.18 ± 0.06b	14.03 ± 0.05a	14.23 ± 0.02b
pH	3.94 ± 0.01c	3.99 ± 0.02d	3.74 ± 0.02a	3.79 ± 0.02b	3.83 ± 0.02b	4.03 ± 0.02d
Urea	1.43 ± 0.01b		1.45 ± 0.02b		0.12 ± 0.04a	0.08 ± 0.01a
Color Intensity	6.16 ± 0.03b	5.38 ± 0.06a	6.29 ± 0.06c	5.51 ± 0.07a	6.42 ± 0.08c	6.88 ± 0.03d
Citric Acid (g/L)	0.22 ± 0.01a	0.03 ± 0.02b	0.24 ± 0.03a	0.04 ± 0.03b	0.23 ± 0.03a	0.22 ± 0.02a

Results represent the mean ± SD for three replicates. Means in the same row with the same letter are not significantly different (*p* < 0.05).

#### 2.2.2. Pyruvic Acid 

The highest levels of pyruvic acid were formed during the first days of fermentation (Figure 2C), except for the KT···SK fermentation where another pyruvic formation peak appeared at day 6. The non-*Saccharomyces* yeast produce occasionally more pyruvic acid and more glycerol, both being derived from the glyceropyruvic pathway [38,39,40]. The maximum pyruvic acid concentrations reached were higher than those recorded in earlier works performed using *Saccharomyces cerevisiae* strains selected for their ability to produce pyruvic acid; these produced only between 60 and 130 mg/L of pyruvic acid [36] compared to the 186.38 mg/L reached in this study (Figure 2C) by KT···SK fermentation and the 337.67 mg/L produced by SK. In fermentations where *Schizosaccharomyces* was involved the pyruvic acid production was the highest. Similar results have been reported before [1]. Higher levels of pyruvic acid could be interesting for red wines because it contributes to the production of highly stable color compounds [32,36].

#### 2.2.3. Alcohol

The alcohol levels varied from 14.03 to 14.56 (% *vol*/*vol*) (Table 1). The sugar consumption can also be used to produce higher amounts of compounds other than ethanol, such as glycerol or pyruvic acid, or to increase the yeast biomass [41,42]. The results obtained showed that fermentations involving non-*Saccharomyces* produced lower ethanol levels. These data agree with other authors who confirmed that some non-*Saccharomyces* types of yeast give lower ethanol yields than *Saccharomyces* [10,17,43,44]. Previous studies showed similar results for *Lachancea thermotolerans* [7] and *Schizosaccharomyces pombe* [22].

#### 2.2.4. SO_2_

The final total SO_2_ levels varied from 44.13 to 58.58 mg/L (Table 1). *Lachancea thermotolerans* fermentations showed lower final concentrations of total SO_2_ than fermentations with SC and SK. The manufacturer (Chr. Hansen, Hørsholm, Denmark) offers it as a way of reducing the risk of presenting H_2_S faults. A possible reason for lower sulfur metabolism could be the lower SO_2_ tolerance reported for several strains of non-*Saccharomyces*.

#### 2.2.5. l-Lactic Acid

Figure 2D shows that *Kluyveromyces thermotolerans* Concerto™ (KT) produced l-lactic acid (Table 1) during alcoholic fermentation. The final l-lactic acid produced by *Lachancea thermotolerans* in this study varied from 2.75 to 2.96 g/L, which clearly influenced the final pH (Table 1). Other authors [6] have also observed significant acidification using mixed cultures of *Lachancea thermotolerans* with the main objective of increasing must acidity. The production of l-lactic is also linked to the viable cell concentration [37]. In this study l-lactic production stopped when the *Lachancea thermotolerans* population started to decrease. The assays performed on malolactic fermentations showed an increase in l-lactic acid of about 0.54 g/L (Table 1). These levels were lower than the cases involving *Lachancea thermotolerans*, due to the low initial level of malic acid in the studied must.

#### 2.2.6. l-Malic Acid

Figure 2E shows a progressive decrease to about 0 g/L in malic acid in all fermentations involving *Schizosaccharomyces pombe*. *Schizosaccharomyces* is the only yeast genus able to reduce efficiently malic acid concentration in must [1] during alcoholic fermentation.

#### 2.2.7. Acetic Acid

Previous experiments with *Lachancea thermotolerans* reported significant reductions in acetic acid content [7,37]. On the other hand *Schizosaccharomyces* has been reported to produce acetic acid concentrations up to 1 g/L as main collateral effect [32]. Nevertheless, nowadays there are strains with reduced collateral effects [36]. The acetic acid levels obtained after alcoholic fermentation varied from of 0.32 to 0.41 g/L (Figure 2F). Those values were not excessive and they did not affect wine quality negatively. After malolactic fermentation took place in fermentations involving *Saccharomyces cerevisiae*, small statistical differences were reported (Table 1).

#### 2.2.8. Biogenic Amines

The final levels of biogenic amines were lower than 2 mg/L (Table 2). This histamine value is considered the lowest level [45]. Fermentations involving *Schizosaccharomyces pombe* showed lower levels than those that where malolactic fermentation was performed (Table 2). The use of *Schizosaccharomyces* is of interest to reduce the possibility of lactic acid bacteria growing by removing malic acid (another nutrient source), thus reducing the risk of biogenic amine [1,46] or ethyl carbamate [47] formation. The urea content of the finished wines was less than 0.2 mg/L (Table 1) for fermentations involving *Schizosaccharomyces pombe*. The reported differences were attributed to the special ability of *Schizosaccharomyces* to metabolize urea [48]. This enzymatic activity also could reduce the initial level of ethyl carbamate precursors [1].

**Table 2:** Biogenic amines analysis of *Saccharomyces cerevisiae* 87 by itself (SC), sequential fermentation with *Saccharomyces cerevisiae* 87 *and Kluyveromyces thermotolerans* CONCERTO™ (KT···SC), sequential fermentation with *Schizosaccharomyces pombe* V2 and *Kluyveromyces thermotolerans* CONCERTO™ (KT…SK), *Schizosaccharomyces pombe* V2 by itself (SK), and fermentations after malolactic fermentation with *Oenococcus oeni* 217 (+ MLF).

Compounds	SC	SC + MLF	KT···SC	KT···SC + MLF	KT···SK	SK
Histamine (mg/L)	0.43 ± 0.02a	1.46 ± 0.06b	0.42 ± 0.04a	1.48 ± 0.15b	0.44 ± 0.04a	0.38 ± 0.02a
Tiramine (mg/L)	0.25 ± 0.01a	0.36 ± 0.04b	0.26 ± 0.02a	0.38 ± 0.06b	0.22 ± 0.03a	0.26 ± 0.03a
Phenylethylamine (g/L)	n.d.	n.d.	n.d.	n.d.	n.d.	n.d.
Putrescine (g/L)	1.78 ± 0.03a	2.18 ± 0.18b	1.82 ± 0.11a	2.24 ± 0.21b	1.71 ± 0.08a	1.88 ± 0.07a
Cadaverine (g/L)	0.51 ± 0.02a	0.65 ± 0.04b	0.49 ± 0.05a	0.69 ± 0.07b	0.52 ± 0.03ab	0.55 ± 0.03a

Results represent the mean ± SD for three replicates. Means in the same row with the same letter are not significantly different (*p* < 0.05).

### 2.3. Sensory Evaluation

During an informal tasting session differences in color, aroma, and taste were found between the wines. No apparent off-flavors were detectable. However, a full sensory analysis is needed to confirm and validate these findings.

## 3. Experimental Section

### 3.1. Microorganisms

The following yeasts were used for the experimental fermentations: *Kluyveromyces thermotolerans* Concerto™ (Hansen, Hørsholm, Denmark; www.chr-hansen.com) that belongs to the yeast species *Lachancea thermotolerans*, *Saccharomyces cerevisiae* 87 (Spanish Type Culture Collection, Valencia, Spain) and *Schizosaccharomyces pombe* V2 (Chemistry and Food Technology department, Polytechnic University of Madrid, Spain [36]). The strain of lactic acid bacteria used was *Oenococcus oeni* 217 (Spanish Type Culture Collection, Valencia, Spain).

### 3.2. Vinification

All fermentations were undertaken using the must of *Vitis vinifera* L. cultivar Tempranillo grapes grown in El Socorro Experimental vineyard (Madrid, Spain). The must was pasteurized at 105 °C for 5 min. A microvinification method similar to those described in scientific literature was used [15,22,32,33,49]. Pasteurized must (4 L) was placed in 5 L glass tanks. This allowed an adequate space for the release of carbon dioxide during the fermentation process. No sulphur dioxide was added to any vessel. Sugar concentration was 249.33 g/L, pH = 3.92, primary amino nitrogen (PAN) 167 g/L, malic acid 0.96 g/L, citric acid 0.24 g/L, lactic and acetic acid bellow 0.1 g/L. To provide nutrition 60 g/hL of Actimax NATURA (Agrovín S.A., Ciudad Real, Spain) were added. Four assays were performed (all in triplicate): (i) inoculation of the must with *S. cerevisiae* 87 (10^6^ CFU/mL) alone (SC); (ii) inoculation of the must with *K. thermotolerans* Concerto™ (10^7^ CFU/mL) followed by *S. cerevisiae* 87 (10^6^ CFU/mL) 96 h later (KT···SC); (iii) inoculation of the must with *K. thermotolerans* Concerto™ (10^6^ CFU/mL) followed by *S. pombe* V2 (10^6^ CFU/mL) 96 h later (KT···SK); and (iv) inoculation of the must with *S. pombe* V2 alone (SK). Yeast inocula were performed using 100 mL of sterilized must with 1 mL of yeast extract dextrose peptone liquid medium [50] containing 10^8^ CFU/mL (determined using a Thomas chamber). To reach this population, 100 μL of each yeast suspension were cultivated in 10 mL of YEPD at 25 °C for 24 h. This procedure was repeated three successive times before the final inoculation of 1 mL in the inocula. All inocula were performed in 250-mL flasks sealed with a Müller valve filled with 98% H_2_SO_4_ (Panreac, Barcelona, Spain), which allowed the release of CO_2_ while avoiding microbial contamination [51]. The temperature was maintained at 25 °C for 48 h. The progress of the inocula was developed under anaerobic conditions. All fermentations were performed in triplicate. All fermentation processes were carried out at 20 °C. When the sugar content was below 3 g/L, the wines were racked and stabilized during 7 days at 4 °C concluding with the final product being bottled. Then a concentration of 50 mg/L of sulphur dioxide in potassium metabisulfite form was added. Sealed bottles were placed horizontally in a climate chamber at 4 °C until the sensory evaluation took place. The wines fermented with *Saccharomyces cerevisiae* by itself (SC), were stabilized and racked following the same procedure, since they finished malolactic fermentation by *Oenococcus oeni* 217 (10^6^ CFU/mL) in 2.8 L vessels at 18 °C. Then they remained under the same final storage conditions described above, for one month before tasting sessions took place.

### 3.3. Analytical Determinations of Non-Volatile Compounds

Glucose and fructose, l-lactic acid, acetic acid, glycerol, pyruvic acid, citric acid, l-malic acid, urea and primary amino nitrogen were all determined using a Y15 enzymatic autoanalyzer (Biosystems S.A, Barcelona, Spain) and its proper kits. Ethanol, pH, free SO_2_, total SO_2_ were determined following the methods in the Compendium of International Methods of Analysis of Musts and Wines [52].

### 3.4. Microvinifications Growth Kinetics

During fermentations, aliquots were taken periodically under aseptic conditions and further seria lten-fold dilutions were made. Yeast growth kinetics were monitored by plating 100 μL of the appropriate dilution on lysine media (non-*Saccharomyces* counts; [53]), YEPD media (total yeast counts; [50]) and YEPDActBzCl media (*Schizosaccharomyces* counts; [35,36]) based on actidione and benzoic acid as main inhibitor agents. In KT···SC fermentations the population of *Lachancea thermotolerans* was estimated by the difference between YEPD and Lysine media counts. In KT···SK fermentations the population of *Lachancea thermotolerans* was estimated by the difference between YEPD and YEPDActBzCl media counts. Colonies were counted after growth at 30 °C for 48–72 h. Lactic bacteria were monitored in MRS agar (Oxoid, Basingstoke, UK).

### 3.5. Analytical Determinations of Biogenic Amines 

The aminoacids were analysed using a Jasco (Tokyo, Japan) UHPLC chromatograph series X-LCTM, equipped with a 3120-FP fluorescence detector. Gradients of solvent A (methanol/acetonitrile, 50:50, *v/v*) and B (sodium acetate /tetrahydrofuran, 99:1, *v*/*v*) were used in a C18 (HALO, city, state abbrev USA) column (100 mm × 2.1 mm; particle size 2.7 µm) as follows: 90% B (0.25 mL/min) from 0 to 6 min, 90%–78% B linear (0.2 mL/min) from 6 to 7.5 min, 78% B from 7.5 to 8 min, 78%–74% B linear (0.2 mL/min) from 8 to 8.5 min, 74% B (0.2 mL/min) from 8.5 to 11 min, 74%–50% B linear (0.2 mL/min) from 11 to 15 min, 50% B (0.2 mL/min) from 15 to 17 min, 50%–20% B linear (0.2 mL/min) from 17 to 21 min, 20%–90% B linear (0.2 mL/min) from 21 to 25 min and re-equilibration of the column from 25 to 26 min. Detection was performed by scanning in the 340–455 nm range. Quantification was performed by comparison against external standards of the studied amines. The different amines were identified by their retention times.

### 3.6. Sensory Evaluation

The experimental wines were evaluated by a team of 15 experienced wine tasters (five females and ten males), all employees of the Chemistry and Food Technology Department (Madrid, Spain) and the Estación Enológica de Haro (Haro, Spain). Two visual descriptors, four taste parameters and five aromas were used to evaluate the final fermentations. No specific training was carried out prior to tasting sessions. Twelve wines were evaluated in randomized order. The wines were presented in clear tasting glasses [54] identified by numbers from 1 to twelve and in an air-conditioned (20 °C) tasting room equipped with individual booths. Twenty five milliliters of each wine were served at 14 °C in randomized order. The panelists were asked to rate typicality regarding their personal Tempranillo wine concept after testing on an unstructured 10 cm scale, from 0 (no defect) to 10 (very strong defect perceptible), to rate the intensity of the 12 attributes. Additionally, the panelists were asked to name descriptors as free comments for each wine.

### 3.7. Statistical Analysis

All statistical analyses were performed using PC Statgraphics v.5 software (Graphics Software Systems, Rockville, MD, USA). The significance was set to *p* < 0.05 for the ANOVA matrix F value. The multiple range test was used to compare the means.

## 4. Conclusions

The comparison of the results from the fermentation trials showed differences in several analyzed parameters. The combination of the non-*Saccharomyces Lachancea thermotolerans* and *Schizosaccharomyces pombe* positively influenced wine quality in the studied case of a low acidic Tempranillo must. Fermentation kinetics showed a fast decline of *Lachancea thermotolerans* yeast immediately after a more fermentative yeast specie was inoculated. All non-*Saccharomyces* fermentations produced higher levels of glycerol and pyruvic acid without increasing acetic acidity. *Lachancea thermotolerans* sulphur dioxide production was significantly lower. All non-*Saccharomyces* produced reduced ethanol levels. The combination of *Lachancea thermotolerans* and a selected *Schizosaccharomyces pombe* strain produced wines stabilised from a malic acid point of view without any need of performing a malolactic fermentation. These wines also showed lower final levels of biogenic amines than the controls that underwent malolactic fermentation.
